# Mycosins of the Mycobacterial Type VII ESX Secretion System: the Glue That Holds the Party Together

**DOI:** 10.1128/mBio.02062-16

**Published:** 2016-12-13

**Authors:** Jeffrey M. Chen

**Affiliations:** Vaccine and Infectious Disease Organization, International Vaccine Centre, University of Saskatchewan, Saskatoon, Saskatchewan, Canada

## Abstract

Since their discovery as important determinants of virulence and growth, the type VII ESX secretion systems (ESX-1 to ESX-5) of slow-growing pathogenic mycobacteria have been the focus of intense scrutiny. Genetic studies have been instrumental in identifying the core components and substrates of these molecular secretion machines and have helped uncover the multifunctional properties of some of them. For instance, the mycosin MycP_1_ of ESX-1, a membrane-associated subtilisin-like serine protease, was shown to have dual functions: the entire protein is essential for ESX-1 function, but only the serine protease regulates secretion activity. MycP_5_ of ESX-5, on the other hand, is required for ESX-5 secretion activity, but the function of its predicted serine protease remains unknown. Recently, van Winden and colleagues (mBio 7:e01471-16, 2016, http://dx.doi.org/10.1128/mBio.01471-16) reported compelling evidence that MycP_1_ and MycP_5_ serve to stabilize the interactions of core ESX-1 and ESX-5 components, respectively, thus explaining how they facilitate the secretion activities of their associated systems.

## COMMENTARY

Mycobacteria have evolved specialized type VII secretion systems to transport molecular cargo across their thick and complex cell envelopes (1). In slow-growing pathogenic mycobacteria, like *Mycobacterium tuberculosis* and *Mycobacterium bovis*, the causative agents of tuberculosis (TB), and *Mycobacterium marinum*, the agent of TB in fish and amphibians and a less hazardous surrogate model often used to study TB pathogenesis, there are five paralogous type VII secretion systems, called ESX-1 to ESX-5 (1). All five share a set of common features, in that the genetic clusters encoding each ESX system contain genes for (i) small secreted proteins of about 100 amino acids with a conserved Trp-X-Gly (WXG) motif located in the middle of the polypeptide (e.g., EsxA of ESX-1, EsxN of ESX-5, etc.), (ii) one putative cell membrane-associated protein (e.g., EccB_1_ of ESX-1, EccB_5_ of ESX-5, etc.), (iii) transmembrane ATPases of the FtsK-SpoIIIE family (e.g., EccCa_1_ and EccCb_1_ of ESX-1, EccC_5_ of ESX-5, etc.), (iv) one 11-transmembrane domain protein that presumably forms a channel in the mycobacterial cell membrane (e.g., EccD_1_ of ESX-1, EccD_5_ of ESX-5, etc.), (v) with the exception of ESX-4, another putative cell membrane-associated protein (e.g., EccE_1_ of ESX-1, EccE_5_ of ESX-5, etc.), and (vi) one subtilisin-like mycosin (e.g., MycP_1_ of ESX-1, MycP_5_ of ESX-5, etc.) (1).

Of the five mycobacterial type VII secretion systems, ESX-1 has been the most studied and is reviewed in excellent detail elsewhere (1). Briefly, ESX-1 in *M. tuberculosis*, *M. bovis*, and *M. marinum* is crucial for the secretion of its associated protein substrates (namely, EsxA, EsxB, EspA, EspB, and EspC) and for mediating virulence through an incredibly diverse number of ways (1). Indeed, it was a spontaneous genetic deletion resulting in the inactivation of ESX-1 in a virulent isolate of *M. bovis* that set it on its inexorable march toward attenuation and the eventual derivation of the live TB vaccine *M. bovis* BCG (1, 2). ESX-3 has been implicated in metal homeostasis and is indispensable for *M. tuberculosis* growth *in vitro* and *in vivo* (1, 3–5). ESX-5 is essential for *M. tuberculosis* and *M. marinum* viability under standard *in vitro* growth conditions and for the secretion of select Pro- and Gln-rich (Pro-Gln [PE] and Pro-Pro-Gln [PPE]) proteins, EsxN, and a posttranslationally cleaved lipase called LipY (1, 6–9). ESX-5 is also involved in modulating the host immune response to *M. tuberculosis* and *M. marinum* (1, 6–9). Meanwhile, ESX-2 and ESX-4 have remained uncharacterized (1).

In the earliest study of *M. tuberculosis* mycosins, beginning in the year 2000, amino acid sequence analysis of its five MycP proteins led to the identification of an Asp-His-Ser catalytic triad also found in bacterial serine proteases of the subtilisin family (10). Moreover, MycP_1_, MycP_2_, and MycP_3_ were shown to be expressed only in the slow-growing *M. bovis* and *M. tuberculosis* species but not in the fast-growing saprophyte *M. smegmatis* (10). In a subsequent study, *M. tuberculosis* MycP_1_ was found to localize to the cell envelope and possess proteolytic activity sensitive to inhibition by serine/cysteine protease inhibitors and activation by Ca^2+^, properties typical of subtilisins (11). Almost a decade later, a *mycP*_*1*_ deletion mutant of *M. tuberculosis* was reported to be defective in ESX-1 secretion activity and attenuated for virulence (12). In addition, the serine-protease domain of MycP_1_ was found to mediate post-translational cleavage of the ESX-1-secreted protein EspB but appeared expendable for overall ESX-1 secretion activity (12). Surprisingly however, the *M. tuberculosis mycP*_*1*_ deletion mutant complemented with and expressing serine protease-dead MycP_1_ displayed ESX-1-mediated hypersecretion (12). Based on these observations, it was concluded that MycP_1_ serves a dual function, with the entire protein being essential for ESX-1 activity and the serine-protease domain being required for cleaving EspB and regulating secretion (12). MycP_5_ was also found to be a functionally integral part of ESX-5, essential for *M. marinum* and *M. bovis* BCG growth and for the secretion of PE/PPE proteins (6). Exactly how MycP_1_ and MycP_5_ facilitate the secretion activities of their respective ESX systems remained poorly understood.

In a recent study reported in *mBio*, van Winden and colleagues presented findings on *M. marinum* MycP_1_ and MycP_5_ that addresses this question (13). The authors first generated a *mycP*_*1*_ deletion mutant of *M. marinum* and found its ESX-1 secretion activity to be completely abolished. Moreover, the *mycP*_*1*_ deletion mutant was unable to mediate ESX-1-dependent lysis of erythrocytes. Complementation of the mutant with *mycP*_1_ restored the wild-type (WT) secretion and virulence phenotype. However, complementation with a serine protease-dead version of *mycP*_*1*_ (*mycP_1_*^S354A^) resulted in the secretion of unprocessed EspB and enhanced ESX-1-mediated secretion and erythrocyte lysis (13). They then went one step further and also complemented the *mycP*_*1*_ deletion mutant with a “bulky” variant of *mycP*_1_ (*mycP_1_*^N239Y^), the construction of which was informed by previous structural work detailing the EspB-binding site of MycP_1_ (14). Replacement of Asn-239 with a larger Tyr residue in the EspB-binding groove of MycP_1_ was predicted to effectively block protease activity. Indeed, complementation with *mycP_1_*^N239Y^ also resulted in unprocessed EspB secretion, ESX-1 substrate hypersecretion, and enhanced erythrocyte lysis. These results, taken together, confirm in *M. marinum* what was previously observed with *M. tuberculosis* MycP_1_: the protein has a dual function with respect to the ESX-1 system.

To determine if the conserved serine protease domain of MycP_5_ plays a similar role in MycP_1_, van Winden et al. complemented a *mycP*_*5*_ deletion mutant of *M. marinum* defective in PE/PPE and EsxN secretion with genes encoding predicted protease-dead (*mycP_5_*^S461A^) and “bulky” (*mycP_5_*^D362Y^) variants of *mycP*_*5*_. Both variant genes restored WT ESX-5 function to the *mycP*_*5*_ deletion mutant, but hypersecretion of PE/PPE and EsxN was not observed. Moreover, differential processing of LipY, a presumed substrate of MycP_5_, was not observed. These results indicate MycP_5_ is required for ESX-5-mediated secretion but, unlike MycP_1_, its serine protease does not appear to be involved in regulating secretion or processing of ESX-5 substrates.

van Winden and colleagues had previously devised a biochemical approach involving *n*-dodecyl beta-d-maltoside (DDM) solubilization of WT *M. marinum* cell envelope proteins, Blue native polyacrylamide gel electrophoresis (PAGE), immunoblotting, and mass spectrometry to show that the core ESX-5 secretion complex is approximately 1,500 kDa in size and contains EccB_5_, EccC_5_, EccD_5_, and EccE_5_ (15). By immunoprecipitating EccB_5_ and pulling down EccC_5_, EccD_5_, and EccE_5_, they confirmed the core ESX-5 secretion complex consists of these interacting proteins (15). Taking the same approach in this study, van Winden et al. resolved DDM-solubilized WT *M. marinum* cell envelope proteins by Blue native PAGE and by immunoblotting identified an EccB_1_-containing complex similar in size (~1,500 kDa) to the core ESX-5 secretion complex. To isolate the putative core ESX-1 complex itself, van Winden et al. complemented an *M. marinum eccCb*_*1*_ transposon mutant with twin Strep-tagged *eccCb*_*1*_. Having verified the Strep tags did not affect EccCb_1_ function by fully complementing the *eccCb*_*1*_ transposon mutant, the authors resolved cell envelope extracts from the complemented mutant by using Blue native PAGE and immunoblotting with Strep antibodies, and identified a corresponding EccCb_1_-containing complex also 1,500 kDa in size. Using Strep-Tactin beads, the tagged EccCb_1_ protein was pulled down along with a number of interacting proteins. Subsequent mass spectrometry analysis revealed these to be EccB_1_, EccCa_1_, EccD_1_, and EccE_1_. The authors also complemented an *eccC*_*5*_-deficient mutant with Strep-tagged *eccC*_*5*_ and pulled down tagged EccC_5_ along with proteins subsequently identified by mass spectrometry to be EccB_5_, EccD_5_, and EccE_5_, as seen previously in immunoprecipitation experiments with EccB_5_ (15). These results, taken together, confirm that the core ESX-1 and ESX-5 secretion complexes in *M. marinum* are similar.

Despite mass spectrometry results that indicated that MycP_1_ and MycP_5_ are not integral components of their respective core secretion complexes, van Winden speculated that the two proteins might still be required for their proper functioning. Comparative Blue native PAGE of cell envelope extracts from WT, *mycP*_*5*_-deficient, and complemented *M. marinum* cells followed by immunoblotting with anti-EccB_5_ antibody revealed the 1,500-kDa core ESX-5 complex to be absent in the *mycP*_*5*_-deficient mutant. Comparison of WT, *mycP*_*1*_-deficient, and complemented *M. marinum* strains also revealed the 1,500-kDa core ESX-1 complex to be absent in the *mycP*_*1*_-deficient mutant. The authors then correctly speculated that MycP_1_ and MycP_5_ may be required to stabilize their respective core secretion complexes. To test this, they pretreated *mycP*_*1*_- and *mycP*_*5*_-deficient *M. marinum* cells with chemical cross-linkers prior to extraction and analysis, working off the assumption that the cross-linkers would help stabilize the core complexes in the absence of MycP_1_ and MycP_5_. Indeed, only upon such treatment were both core ESX-1 and ESX-5 membrane complexes stabilized at WT levels. These results taken together indicate that MycP_1_ and MycP_5_ facilitate the secretion activities of ESX-1 and ESX-5, respectively, by stabilizing their core secretion complexes.

This study by van Winden and colleagues is important for several reasons. First, the authors have confirmed in another slow-growing pathogenic mycobacterium that MycP_1_ does indeed perform dual roles. However, unlike MycP_1_, MycP_5_ in *M. marinum* at least does not appear to share the same dual-function property, even though it has a conserved serine-protease domain. This may well be a reflection of or an underlying reason for the functional differences between the ESX-1 and ESX-5 systems in mycobacterial biology. Further studies to identify the substrates for MycP_5_ and ascertain the role of its serine-protease domain are warranted. Additional studies should also be done to determine the consequences for *M. marinum* expressing protease-dead MycP_1_ in host-pathogen interactions, given that in *M. tuberculosis*, ESX-1 hypersecretion appears to compromise the bacterium’s virulence in chronic infections (12). Second, the authors have clearly demonstrated that the core ESX-1 complex, at least in *M. marinum*, is composed of EccB_1_, EccCa_1_, EccCb_1_, EccD_1_, and EccE_1_. Efforts should now focus on assessing these complexes in either *M. tuberculosis*, *M. bovis*, or recombinant *M. bovis* BCG with a restored ESX-1 system (e.g., BCG::RD1). Third, the authors addressed how MycP_1_ and MycP_5_ facilitate secretion by showing that the proteins help stabilize the formation of their respective core secretion complexes. The same approaches used by these investigators might also be valuable in determining if ESX-1-associated proteins, such as EspA, EspC, EspD, and EccA_1_, are also needed to stabilize the core ESX-1 secretion complex and, in doing so, enable secretion. Finally, the van Winden et al. study nicely demonstrates how the judicious use of ESX mutants in combination with biochemical approaches can yield further insights into the functional and temporal organization of these incredibly dynamic molecular machines. Experimental schemes using these approaches will surely reveal more details about the mycobacterial ESX systems in the future.
